# Association Between Serum Magnesium and Glycemic Control, Lipid Profile and Diabetic Retinopathy in Type 1 Diabetes

**DOI:** 10.7759/cureus.21128

**Published:** 2022-01-11

**Authors:** Isabel Inácio, Teresa Azevedo, Ana Margarida Balsa, Sara Ferreira, Patrícia Rosinha, Márcia Alves, Rosa Dantas, Joana Guimarães

**Affiliations:** 1 Endocrinology Department, Centro Hospitalar do Baixo Vouga, Aveiro, PRT; 2 Endocrinology Department, Centro Hospitalar de Trás-Os-Montes E Alto Douro, Vila Real, PRT

**Keywords:** hypomagnesemia, dr, mg, type 1 diabetes, magnesium, diabetic retinopathy, dyslipidemia, glycemic control, serum magnesium, type 1 diabetes mellitus (t1dm)

## Abstract

Introduction: Many studies have shown an association between decreased serum magnesium (Mg) levels and poor glycemic control and dyslipidemia in individuals with type 1 diabetes (T1D). Few studies evaluated the association between magnesium (Mg) levels and diabetic retinopathy (DR) in individuals with type 1 diabetes (T1D).

Methods: Retrospective study of adults with T1D, with an ophthalmological evaluation and a serum Mg level determination. According to Mg levels, the individuals were stratified into two groups: normal Mg levels (1.81-2.60 mg/dL) and low Mg levels (≤1.80 mg/dL). Exclusion criteria were individuals on diuretics or proton-pump inhibitors, malabsorption or diarrhea, oral magnesium supplementation in the recent past, pregnancy, or sepsis.

Results: 105 individuals, with median Mg levels of 1.96 (interquartile range 0.23) mg/dL. Hypomagnesemia (≤1.80 mg/dL) was detected in 20.0% individuals and 26.7% had DR. Individuals with hypomagnesemia had higher HbA1c (p = 0.014) and triglycerides (p = 0.024). Mg levels were negatively correlated with systolic blood pressure (r = -0.200, p = 0.041), HbA1c (r = -0.281, p = 0.004) and body mass index (BMI) (r = -0.197, p = 0.041). There was no significant difference between Mg levels or prevalence of hypomagnesemia in individuals with or without DR. Also, there was no significant difference between Mg levels and the severity of DR.

Conclusion: Hypomagnesemia is a common problem in adults with T1D, and it was correlated with poor glycemic control, although we did not find a significant association between Mg levels and prevalence or severity of DR.

## Introduction

Many studies have shown an association between decreased serum magnesium (Mg) levels and poor glycemic control and dyslipidemia in individuals with type 1 diabetes (T1D), according to a recent systematic review and meta-analysis [1]. However, few studies have evaluated the association between reduced Mg levels and diabetes complications in these patients and, in particular with diabetic retinopathy (DR), divergent results were found [2-4].

In this recent systematic review [1], one study that was included found no association between reduced Mg levels and DR [3], while another [4] showed an association between increased Mg levels and DR. Previously, a systematic review that included individuals with T1D and type 2 diabetes also found that daily intake of Mg levels did not appear to be associated with DR [5].

This study aims to evaluate the status of serum Mg levels in adults with T1D and assess the association between Mg levels and glycemic control, lipid profile, and DR.

This study was previously presented as a meeting abstract at the endocrine society annual business meeting (ENDO) 2021 on March 20-23, 2021.

## Materials and methods

This retrospective study was carried out in the endocrinology department of Centro Hospitalar do Baixo Vouga in Portugal. Adults (≥ 18 years) with T1D with an ophthalmological evaluation and a serum Mg level determination were included.

The individuals were stratified into two groups according to Mg levels: normal Mg levels (1.81-2.60 mg/dL) and low Mg levels (≤1.80 mg/dL). Exclusion criteria were individuals on diuretics or proton-pump inhibitors, malabsorption or diarrhea, oral magnesium supplementation in the recent past, pregnancy, or sepsis.

According to the recommendations of the European Society of Cardiology (ESC)/European Association for the Study of Diabetes (EASD) [6] and ESC/European Atherosclerosis Society (EAS) [7], dyslipidemia was defined if at least one parameter related to the lipid profile was altered (total cholesterol >190 mg/dL, low-density lipoprotein cholesterol [LDL-C] ≥100 mg/dL, non-high-density lipoprotein cholesterol [non-HDL-C] ≥130 mg/dL, triglycerides (TG) ≥150 mg/dL. Hypertension was considered if previous diagnosis or under antihypertensive medication.

Statistical analysis was performed using the IBM Corp. Released 2019. IBM SPSS Statistics for Windows, Version 26.0. Armonk, NY: IBM Corp. Categorical variables are presented as frequencies and percentages and continuous as median (interquartile range). Fisher’s exact, Mann-Whitney and Kruskal-Wallis tests and Spearman's correlation were used. A value of p <0.05 was considered statistically significant.

## Results

We evaluated 105 individuals with T1D (56.2% male) with median age of 36.0 (interquartile range 16.0) years and median T1D duration of 16.0 (12.0) years. Median HbA1c was 7.6 (1.5) percent and median Mg levels were 1.96 (0.23) mg/dL. Hypomagnesemia (Mg ≤1.80 mg/dL) was detected in 20.0% (n=21) individuals and 26.7% (n=28) had DR. Individuals with hypomagnesemia had higher HbA1c [8.2 (1.6) vs. 7.5 (1.3) percent, p=0.014] and triglycerides [97.0 (43.0) vs. 74.0 (53.0) mg/dL, p=0.024]. There was no statistical difference in prevalence of DR between the two groups stratified by Mg levels (Table 1).

**Table 1:** Clinical and biochemical characteristics of adults with type 1 diabetes according to serum magnesium levels. Data are presented as median (interquartile range) or as n (%) unless otherwise indicated. BMI: body mass index; eGFR: estimated glomerular filtration rate; HbA1c: hemoglobin A1c; HDL-C: high-density lipoprotein cholesterol; Mg: magnesium; LDL-C: low-density lipoprotein cholesterol.

	Total (n=105)	Low Mg levels (≤1.80 mg/dL) (n=21)	Normal Mg levels (1.81-2.60 mg/dL) (n=84)	*p* value
Male, n (%)	59 (56.2)	9 (42.9)	50 (59.5)	0.220
Age, years	36.0 (16.0)	36.0 (12.0)	35.0 (16.0)	0.962
T1D duration, years	16.0 (12.0)	17.0 (11.0)	16.0 (13.0)	0.813
Diabetic Retinopathy, n (%)	28 (26.7)	7 (33.3)	21 (25.0)	0.424
eGFR <60 mL/min/1.73m^2^, n (%)	5 (4.8)	1 (19.0)	4 (4.8)	0.735
Dyslipidemia, n (%)	49 (46.7)	10 (47.6)	39 (46.4)	0.557
Hypertension, n (%)	25 (23.8)	8 (38.1)	17 (20.2)	0.079
Systolic blood pressure, mmHg	129.0 (19.0)	133.0 (19.0)	128.0 (20.0)	0.297
Diastolic blood pressure, mmHg	81.0 (14.0)	81.0 (10.0)	81.0 (15.0)	0.453
BMI, kg/m^2^	25.4 (5.6)	26.6 (7.7)	25.0 (5.0)	0.095
Mg, mg/dL	1.96 (0.23)	1.74 (0.14)	1.99 (0.18)	<0.001
HbA1c, %	7.6 (1.5)	8.2 (1.6)	7.5 (1.3)	0.014
Total cholesterol, mg/dL	180.0 (44.0)	166.0 (44.3)	181.0 (41.2)	0.080
HDL-C, mg/dL	61.2 (26.6)	57.3 (18.3)	61.9 (27.9)	0.185
LDL-C, mg/dL	107.5 (38.3)	101.0 (48.5)	108.5 (36.5)	0.341
Triglycerides, mg/dL	76.0 (55.0)	97.0 (43.0)	74.0 (53.0)	0.024
25-hydroxyvitamin D, ng/mL	14.6 (9.8)	13.1 (10.5)	15.0 (9.3)	0.286

Mg levels were negatively and weakly correlated with systolic blood pressure (r = -0.200, p = 0.041), HbA1c (r = -0.281, p = 0.004) and body mass index (BMI) (r = -0.197, p = 0.041). There was no correlation between Mg levels and total cholesterol (r = 0,144; p = 0,142), low-density lipoprotein cholesterol (r = 0,041; p = 0,677), non-high-density lipoprotein cholesterol (r = 0,112; p = 0,254) or triglycerides (r = -0,109; p = 0,267).

There was no significant difference between Mg levels [1.96 (0.28) vs. 1.96 (0.19) mg/dL, p = 0.986] or prevalence of hypomagnesemia [25% vs. 18.4%, p = 0.424] in patients with or without DR (Table 2).

**Table 2:** Clinical and biochemical characteristics of adults with type 1 diabetes according to the presence or absence of diabetic retinopathy. Data are presented as median (interquartile range) or as n (%) unless otherwise indicated. BMI: body mass index; DR: diabetic retinopathy; eGFR: estimated glomerular filtration rate; HbA1c: hemoglobin A1c; HDL-C: high-density lipoprotein cholesterol; Mg: magnesium; LDL-C: low-density lipoprotein cholesterol.

	DR (n=28)	No DR (n=77)	*p* value
Male, n (%)	19 (67.9%)	40 (51.9%)	0.184
Age, years	39.5 (12.0)	33.0 (15.0)	0.025
T1D duration, years	22.0 (9.0)	13.0 (12.0)	<0.001
eGFR <60 mL/min/1.73m^2^, n (%)	3 (10.7%)	2 (2.6%)	0.120
Dyslipidemia, n (%)	16 (57.1%)	33 (42.9%)	0.269
Hypertension, n (%)	12 (42.9%)	13 (16.9%)	0.009
Systolic blood pressure, mmHg	136.5 (27.0)	127.0 (20.0)	0.007
Diastolic blood pressure, mmHg	85.0 (12.0)	78.0 (13.0)	0.001
BMI, kg/m^2^	26.6 (4.6)	24.7 (5.4)	0.095
Mg, mg/dL	1.96 (0.28)	1.96 (0.19)	0.986
Hypomagnesemia, n (%)	7 (25.0%)	14 (18.4%)	0.424
HbA1c, %	8.1 (1.6)	7.5 (1.4)	0.107
Total cholesterol, mg/dL	180.0 (44.1)	180.0 (38.9)	0.184
HDL-C, mg/dL	57.0 (23.8)	63.8 (31.1)	0.625
LDL-C, mg/dL	109.0 (39.0)	107.0 (32.0)	0.128
Triglycerides, mg/dL	76.0 (68.0)	79 (53.0)	0.466
25-hydroxyvitamin D, ng/mL	12.9 (9.0)	14.7 (9.8)	0.205

There was no statistically significant association between Mg levels and the severity of DR (Table 3) or T1D duration (Table 4).

**Table 3:** Magnesium levels according to severity of DR. Data are presented as median (interquartile range). DR: diabetic retinopathy; Mg: magnesium; NPDR: non-proliferative diabetic retinopathy; PDR: proliferative diabetic retinopathy.

	No DR (n=77)	Minimal NPDR (n=16)	Moderate to severe NPDR (n=4)	PDR (n=4)	Non-stratified DR (n=5)	*p* value
Mg, mg/dL	1.96 (0.19)	1.96 (0.29)	1.97 (0.30)	1.79 (0.38)	2.08 (0.51)	0.665

**Table 4:** Magnesium levels according to type 1 diabetes duration (<20 vs ≥20 years). Data are presented as median (interquartile range). Mg: magnesium; T1DM: type 1 diabetes mellitus.

	T1D duration <20 years (n=67)	T1D duration ≥20 years (n=38)	*p* value
Mg, mg/dL	1.96 (0.21)	1.97 (0.26)	0.934

## Discussion

In our study, we found that hypomagnesemia is a common problem in adults with T1D. Mg levels were correlated with poor glycemic control, although we did not find a significant association between Mg levels and lipid profile other than triglycerides or prevalence or severity of DR.

Our results support the previous data regarding the association between hypomagnesemia and poor glycemic control in patients with T1D. It is still unclear whether hypomagnesemia results in poor glycemic control or vice-versa [1]. However, one study has shown that hyperglycemia increases renal Mg excretion in patients with T1D, suggesting that poor glycemic control can cause hypomagnesemia [8].

Regarding dyslipidemia, we found only a significant association between hypomagnesemia and higher triglycerides, although its levels were within the normal range. Previous studies also found a relation with elevated triglycerides, but also with total cholesterol and LDL-C and reduced HDL-C in T1D [9,10]. It is known that hypomagnesemia may contribute to lower insulin secretion, which may affect lipid metabolism leading to dyslipidemia [11,12].

Mg levels were not related to the presence and the severity of DR in our study, as shown by some previous studies [3, 5,13]. Specifically, regarding the severity of DR, our study is in agreement with the findings of Corrêa et al., suggesting that serum Mg does not seem to influence the severity of DR [13]. In our study, patients with DR were older and had longer T1D duration as expected and also tended to present higher HbA1c levels (Table 2), although we did not find a significant association (p=0.107), probably due to the small population sample size. Future longitudinal studies may elucidate the causality between reduced Mg levels and poor glycemic control, dyslipidemia, and the presence of DR [1].

Our study has a few limitations, namely its small sample size of the population and the lack of follow-up of patients. However, we point out that this study has a larger sample size than previous studies that evaluated the association between reduced Mg levels and DR included in a recent systematic review [2-4]. Thus, we emphasize the importance of conducting studies considering people with this specific type of diabetes and suggest further studies on a larger population and with some follow-up in adults with T1D.

## Conclusions

In the current study, we assessed the status of serum Mg levels and the association between serum Mg levels and glycemic control, lipid profile, and DR in adults with T1D in a Portuguese center. Our results showed that hypomagnesemia is a common problem in adults with T1D and Mg levels were associated with poor glycemic control and higher triglycerides, although we did not find a significant association between Mg levels and other lipid parameters or prevalence or severity of DR. We suggest that it is important to correct hypomagnesemia in T1D in clinical practice and to find out whether oral Mg supplementation can improve glycemic control and lipid profile or affect the presence and severity of DR in T1D in future studies.

## References

[1] Rodrigues AK, Melo AE, Domingueti CP (2020). Association between reduced serum levels of magnesium and the presence of poor glycemic control and complications in type 1 diabetes mellitus: a systematic review and meta-analysis. Diabetes Metab Syndr.

[2] Fort P, Lifshitz F (1986). Magnesium status in children with insulin-dependent diabetes mellitus. J Am Coll Nutr.

[3] Gunn IR, Burns E (1987). Plasma ultrafiltrable magnesium in insulin dependent diabetics. J Clin Pathol.

[4] Walter RM Jr, Uriu-Hare JY, Olin KL, Oster MH, Anawalt BD, Critchfield JW, Keen CL (1991). Copper, zinc, manganese, and magnesium status and complications of diabetes mellitus. Diabe Car.

[5] Lee CT, Gayton EL, Beulens JW, Flanagan DW, Adler AI (2010). Micronutrients and diabetic retinopathy: a systematic review. Ophthalmology.

[6] Cosentino F, Grant PJ, Aboyans V (2020). 2019 ESC Guidelines on diabetes, pre-diabetes, and cardiovascular diseases developed in collaboration with the EASD. Eur Heart J.

[7] Mach F, Baigent C, Catapano AL (2020). 2019 ESC/EAS guidelines for the management of dyslipidaemias: lipid modification to reduce cardiovascular risk. Eur Heart J.

[8] Djurhuus MS, Skøtt P, Vaag A, Hother-Nielsen O, Andersen P, Parving HH, Klitgaard NA (2000). Hyperglycaemia enhances renal magnesium excretion in type 1 diabetic patients. Scand J Clin Lab Invest.

[9] Shahbah D, El Naga AA, Hassan T (2016). Status of serum magnesium in Egyptian children with type 1 diabetes and its correlation to glycemic control and lipid profile. Medicine (Baltimore).

[10] Shahbah D, Hassan T, Morsy S (2017). Oral magnesium supplementation improves glycemic control and lipid profile in children with type 1 diabetes and hypomagnesaemia. Medicine (Baltimore).

[11] Manaswini N, Noorjahan M, Baba KSSS (2017). Hypomagnesemia as a link to dyslipidemia in diabetes mellitus. IOSR J Den Med Sci.

[12] Sociedade Brasileira de Diabetes (2015-2016). Epidemiologia e prevenção da diabetes mellitus: diretrizes da SBD. http://www.diabetes.org.br/profissionais/images/docs/DIRETRIZES-SBD-2015-2016.pdf.

[13] Corrêa ZMS, Freitas AM, Marcon IM (2003). Risk factors related to the severity of diabetic retinopathy. Arquivos Brasileiros de Oftalmologia.

